# An Aerosol-Assisted Chemical Vapor Deposition Route to Tin-Doped Gallium Oxide Thin Films with Optoelectronic Properties

**DOI:** 10.1021/acsaelm.4c00973

**Published:** 2024-08-12

**Authors:** Ruizhe Chen, Sanjayan Sathasivam, Joanna Borowiec, Claire J Carmalt

**Affiliations:** † Materials Chemistry Centre, Department of Chemistry, University College London, 20 Gordon Street, London WC1H 0AJ, U.K.; ‡ School of Engineering, London South Bank University, London SE1 0AA, U.K.

**Keywords:** Thin film, transparent conducting oxides (TCOs), gallium oxide (Ga_2_O_3_), chemical vapor deposition (CVD), dopants

## Abstract

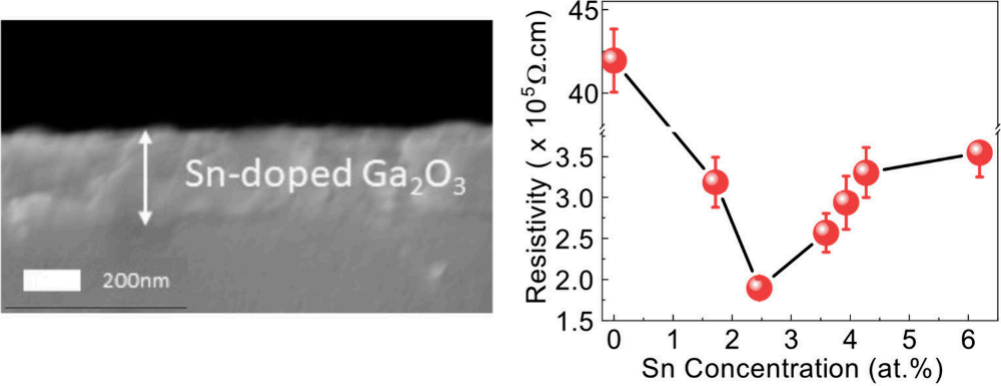

Gallium oxide is a wide-bandgap compound semiconductor material renowned for its diverse applications spanning gas sensors, liquid crystal displays, transparent electrodes, and ultraviolet detectors. This paper details the aerosol assisted chemical vapor deposition synthesis of tin doped gallium oxide thin films using gallium acetylacetonate and monobutyltin trichloride dissolved in methanol. It was observed that Sn doping resulted in a reduction in the transmittance of Ga_2_O_3_ films within the visible spectrum, while preserving the wide bandgap characteristics of 4.8 eV. Furthermore, Hall effect testing revealed a substantial decrease in the resistivity of Sn-doped Ga_2_O_3_ films, reducing it from 4.2 × 10^6^ Ω cm to 2 × 10^5^ Ω cm for the 2.5 at. % Sn:Ga_2_O_3_ compared to the nominally undoped Ga_2_O_3_.

## Introduction

Gallium oxide (Ga_2_O_3_) is an emerging semiconductor material with application in power electronics, in photodetectors, and as transparent conducting oxides (TCOs).^1,2^ Power devices founded upon β-Ga_2_O_3_ exhibit enhanced breakdown voltage and reduced on-resistance. Consequently, these devices result in diminished conduction loss and increased power conversion efficiency.^3−9^ Moreover, devices made from Ga_2_O_3_ can operate at elevated temperatures, thus, mitigating the necessity for voluminous cooling mechanisms. The enhanced thermal performance stems from Ga_2_O_3_’s capacity to endure augmented electric fields without undergoing breakdown, a capability that surpasses that of traditional materials such as silicon, SiC, and GaN.^3,10^

Ga_2_O_3_ also displays potential for power distribution systems in electric vehicle charging infrastructure or converters channeling energy from renewable sources such as wind turbines into the grid.^11−14^ Furthermore, Ga_2_O_3_ emerges as a promising candidate for metal oxide semiconductor field effect transistors (MOSFETs), an electronic component ubiquitous in devices such as laptops and smartphones.^15,16^ Ga_2_O_3_’s viability extends to applications necessitating MOSFETs capable of operating at power levels surpassing the capabilities of traditional silicon-based devices.^1^

Ga_2_O_3_ thin films are also emerging as TCOs due to their ultrawide band gap of 4.8 eV and dopability with higher valence species such as Sn or Si to achieve enhanced conductivity.^17^ TCOs are typically used in solar cells, flat panel displays, organic light-emitting diodes, specialized window coatings, transparent thin film transistors, and flexible electronic devices.^18−23^ Ga_2_O_3_-based TCOs are particularly useful as electrodes for deep UV optoelectronic devices, such as lasers, LEDs, and detectors.^24^

The common methods for synthesizing Ga_2_O_3_ films include physical vapor deposition, chemical vapor deposition, solution deposition methods, pulsed laser deposition, and sputtering.^25−28^ However, this study employs the aerosol-assisted chemical vapor deposition (AACVD) method to prepare Sn-doped Ga_2_O_3_ films. AACVD stands as a modification of the conventional CVD technique.^29−33^ In this process, the precursor is initially dissolved in a solvent. Subsequently, this mixture of the precursor and solvent is transformed into a mist through an ultrasonic humidifier. The resulting mist is then introduced into the reactor via a carrier gas. In certain chemical reactions, the precursors undergo decomposition at elevated temperatures and the ensuing intermediates are simultaneously deposited onto the substrate.

In comparison to the aforementioned synthesis methods, AACVD offers advantages such as low cost, simple equipment and operation, a wide range of precursor compatibility, and convenient multimaterial doping. Basharat et al. have previously produced homogeneous and stable Ga_2_O_3_ films for gas sensing applications using AACVD.^34^ Uno et al. have studied the growth mechanism of Ga_2_O_3_ on sapphire substrates using mist CVD. This study investigates the use of AACVD to deposit doped Ga_2_O_3_ films to enhance electronic conductivity.

## Experimental Section

### Film Synthesis

All precursor materials were procured from Aldrich and utilized without further purification. The AACVD depositions were conducted within a custom-designed cold-wall reactor.^35^ In this setup, a quartz substrate measuring approximately 1 cm × 1 cm was placed on a glass substrate, while a graphite block, housing a Watlow cartridge heater regulated by a Pt–Rh cartridge heater, was positioned for controlled heating. To ensure laminar flow, a stainless-steel top plate was situated 0.8 cm above the substrate.

The Sn-doped Ga_2_O_3_ films were grown via an AACVD process, employing butyltin trichloride at varying concentrations (0, 0.5, 1, 2, 3, 4, and 5 mol %) and Ga(acac)_3_ (0.3 g) dissolved in commercial dry methanol (20 mL, 788 mmol). The precursor solution was atomized using a Johnson Matthey Liquifog piezoelectric ultrasonic humidifier, with the precursor flow rate maintained at 0.5 L min^–1^ by nitrogen (BOC, 99.99%). The quartz substrate was maintained at a temperature of 450 °C throughout the deposition process.

Upon completion of deposition, the reactor was powered off and gradually cooled under a continuous flow of nitrogen until it reached 100 °C. At this juncture, the samples were carefully removed. Subsequently, the coated substrates were transferred to a tube furnace for heat treatment. The film and substrate were annealed in air at 1000 °C for a duration of 12 h.

### Instrumental Conditions

X-ray diffraction (XRD) analysis was conducted using a PANalytical Empyrean system in grazing incidence mode with monochromated Cu Kα radiation. The incident beam angle was set at 0.5°, and the 2θ range of 5–80° was recorded with a step size of 0.05° at 1 s per step. Scanning electron microscopy (SEM) measurements were carried out by utilizing a JEOL JSM-6301F field emission SEM with a 5 keV accelerating voltage. To mitigate charging effects, the samples were coated with a layer of gold. X-ray photoelectron spectroscopy (XPS) was performed by using a Thermo Scientific Kα photoelectron spectrometer, which employed monochromatic Al Kα radiation. Higher resolution scans were acquired for the primary peaks of Sn(3d), Ga(3d), O(1s), and C(1s) with a pass energy of 50 eV. CasaXPS software was utilized for peak fitting, and binding energies were adjusted to adventitious carbon (284.8 eV) for charge correction. For resistivity (ρ) determination, Hall effect measurements were conducted by employing the van der Pauw method using a Ecopia HMS-3000 instrument.

## Results and Discussion

**Figure 1:**
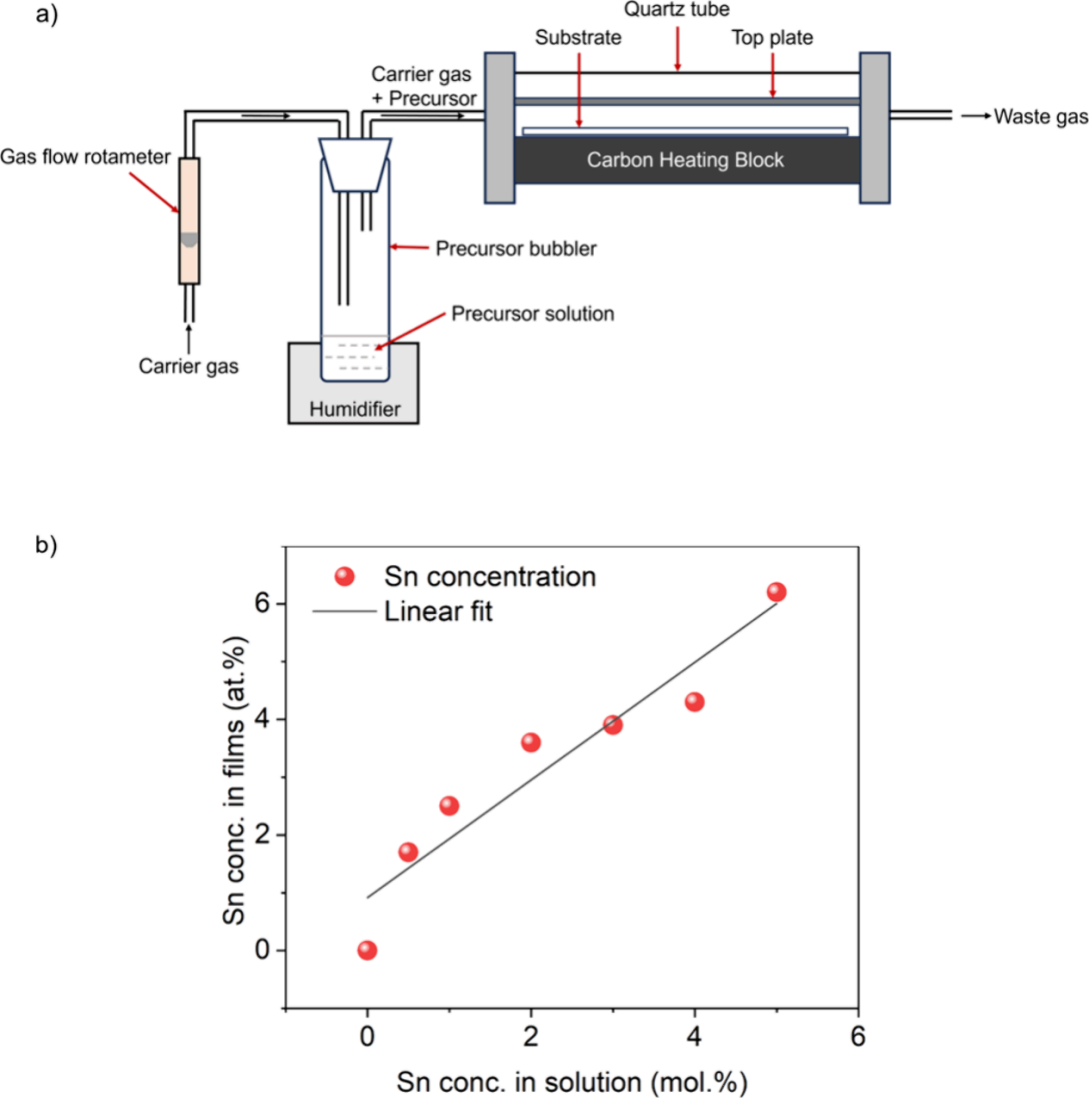
(a) Schematic diagram showing the process involved during aerosol assisted chemical vapor deposition (AACVD). (b) The linear relationship between the amount of MBTC in solution and the Sn concentration obtained in the doped Ga_2_O_3_ thin films grown via AACVD.

Nominally pure and Sn-doped Ga_2_O_3_ thin films were grown on quartz substrates from the AACVD reaction of gallium acetylacetonate (Ga(acac)_3_), methanol, and monobutyltin trichloride (MBTC) at 450 °C (Figure 1a). The oxygen source for the films is thought to come from residual water in the methanol or the methanol itself and not directly from the breakdown of the oxygen containing acetylacetonate moiety of the organometallic precursor used for AACVD.^36^ The concentration of MBTC was varied from 0, 0.5, 1, 2, 3, 4, and 5 mol % relative to Ga(acac)_3_ to obtain Sn concentration of 0, 1.7, 2.5, 3.6, 3.9, 4.3, and 6.2 at. % in the films. This dopant concentration range was appropriate to study the impact of the Sn on the optoelectronic and material properties of the Ga_2_O_3_ thin films including the solubility limit of Sn in the Ga_2_O_3_ lattice. The atomic concentration in the film is largely increased with increasing concentration of the MBTC precursor in the AACVD, suggesting good compatibility with respect to the decomposition of both precursors during the CVD reaction (Figure 1b).

**Figure 2:**
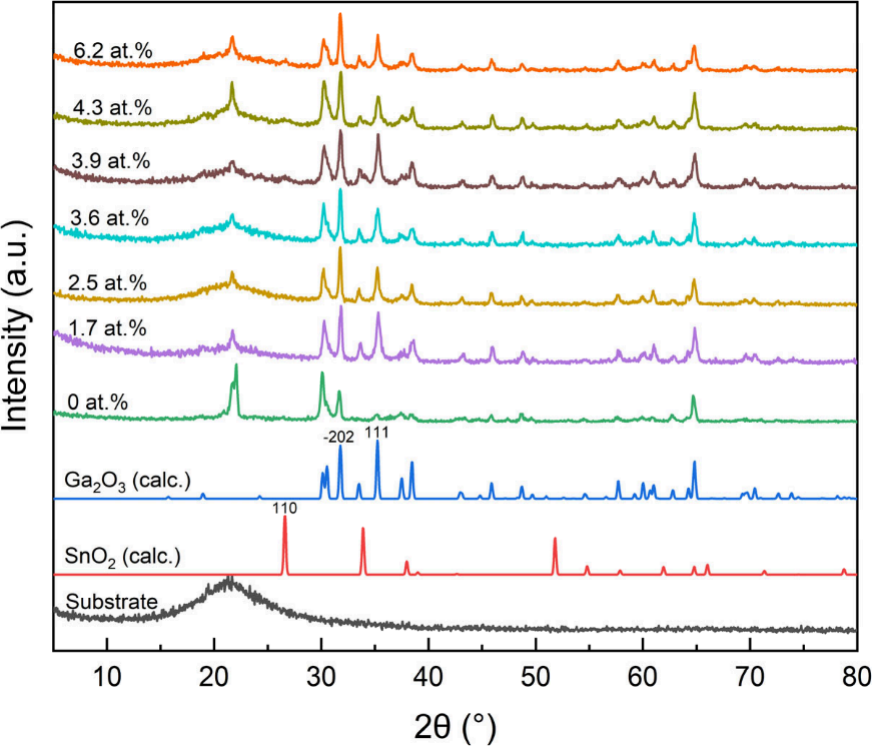
XRD patterns for the 0–6.2 at. % Sn-doped Ga_2_O_3_ films and quartz substrate. The calculated patterns for Ga_2_O_3_ and SnO_2_ are also shown.

Figure 2 displays the X-ray diffraction (XRD) patterns comprising of the calculated profiles for SnO_2_ (cassiterite) and Ga_2_O_3_ (monoclinic), as well as patterns for Sn-doped Ga_2_O_3_ samples with Sn concentrations ranging from 0 to 6.2 at. % and the quartz substrate. The diffraction peak at approximately 22.4° is correlated to the SiO_2_ substrate. For the 0–3.6 at. % doped samples, only peaks matching Ga_2_O_3_ were observed, indicating the successful formation of a solid solution. At the higher dopant concentrations of 3.9 to 6.2 at. %, a peak at 26.7° associated with the (110) plane of SnO_2_ cassiterite becomes visible suggesting that the solubility limit of Sn in Ga_2_O_3_ has been reached and phase separation is taking place. The study by Guillermo et al. also addresses this issue, as the ionic radius of Sn^4+^ ions (0.69 Å) is larger (11.3%) than that of Ga^3+^ ions (0.62 Å), substitutional doping induces lattice distortion that can eventually lead to precipitation of a secondary phase from the solid solution.^37^ In our CVD study, the solubility limit was visibly reached at 2.5 at. % Sn whereas other methods such as sputtering process and hydrothermal synthesis have reported Sn-doped Ga_2_O_3_ nanostructures with the solid solubility of around 1 atom % and 2.2 at. %, respectively.^27,38^

The incorporation of Sn as a dopant induces varied growth orientations in Ga_2_O_3_ films in comparison to the pristine Ga_2_O_3_ films, notably influencing the (−202) and (111) crystallographic directions.^39^ Past studies have offered various explanations for the variation observed in diffraction peaks within X-ray diffraction (XRD) patterns with a predominant focus on the synthesis temperature of the films. Among the distinct structures of Ga_2_O_3_, β-Ga_2_O_3_ demonstrates stability at elevated temperatures, while α- and γ-Ga_2_O_3_ tend to crystallize more effectively at lower temperatures. Moreover, depending on the synthesis method employed and the substrate material chosen, there is a possibility of forming amorphous Ga_2_O_3_.^40−42^ In contrast to the prevalent selection of silicon, β-Ga_2_O_3_, or sapphire as substrate materials in most investigations, the utilization of quartz substrates in this experiment facilitates Ga_2_O_3_ growth without predisposing it to a preferred tendency for epitaxial growth. A similar situation occurs in experiments where In_2_O_3_ is doped with different elements.^43^ It is likely due to the change in surface energy and lattice parameter brought on by dopant atoms.

The full width at half-maximum (fwhm) of the XRD peaks decreased with increasing Sn concentration up to 2.5 at. %, indicating an increase in crystallinity and crystallite size. With further increase in dopant concentration, the fwhm increased again to values close to what was observed for the undoped Ga_2_O_3_ film (Table S1). Interestingly, the presence of the SnO_2_ (110) peak becomes apparent as the Sn doping surpasses 2.5 at%. This shift signifies a transition from Sn-doped Ga_2_O_3_ to a composite of SnO_2_/Ga_2_O_3_ at higher Sn concentrations.

The discernible alterations in the XRD diffraction peaks correspond to shifts in the film crystalline quality. In effect, marginal Sn doping contributes to the enhancement of Ga_2_O_3_ film crystalline quality. Yet, elevated Sn doping concentrations can lead to potential lattice disruption within the Ga_2_O_3_ films due to excessive Sn atom incorporation, potentially precipitating the formation of new phases (e.g., SnO_2_). This occurrence subsequently diminishes the crystalline quality of the Sn-doped Ga_2_O_3_ films.^20^

**Figure 3:**
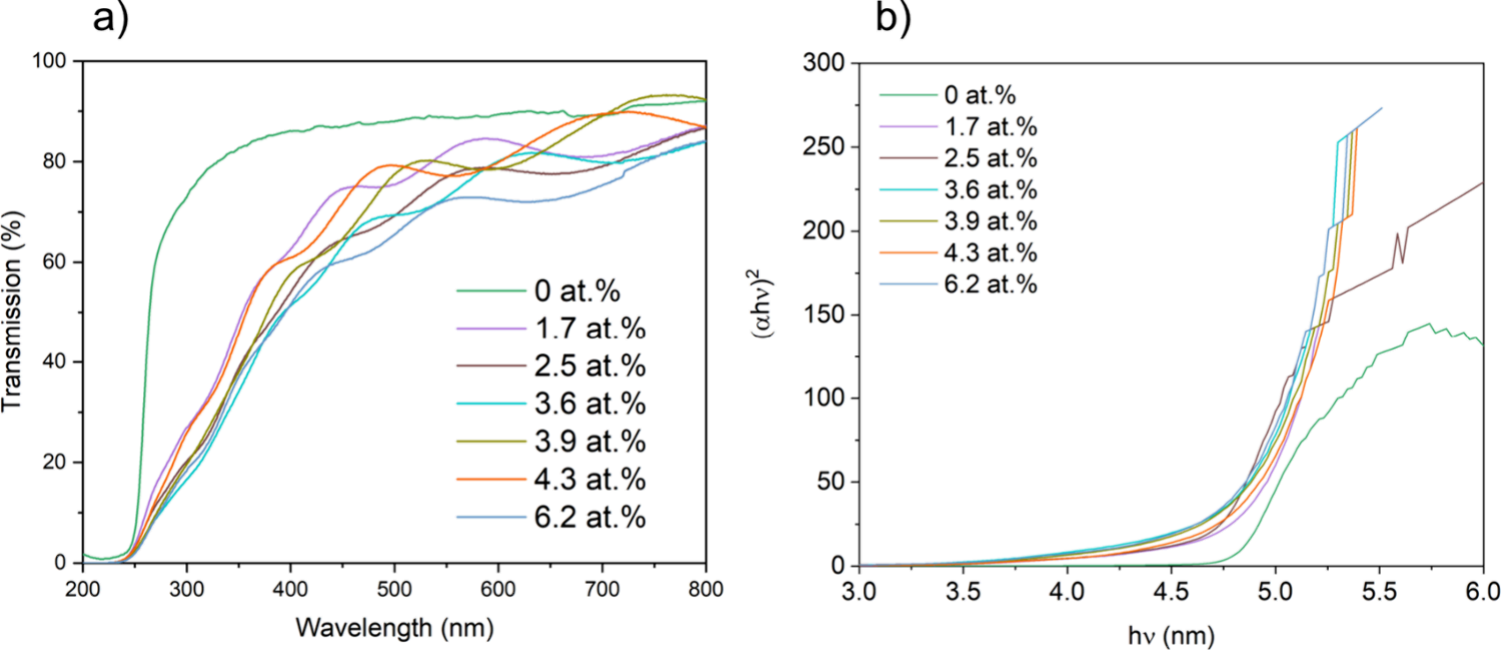
(a) Transmittance spectra of the undoped and Sn-doped Ga_2_O_3_ films grown on quartz substrates and (b) calculated Tauc plot indicating the direct band gap energies.

Figure 3 depicts the transmission spectra of Sn-doped Ga_2_O_3_ films, delineating their response to varying Sn-doping concentrations. These samples exhibit a consistent average transmittance spanning 60% to 85% across both visible wavelength domains. Sn-doped Ga_2_O_3_ films show reduced transmittance in the visible spectrum compared with undoped films. Furthermore, an increase in the Sn doping content is correlated with an intensified light absorption propensity of the Ga_2_O_3_ films, particularly evident within the ultraviolet wavelength range (200–400 nm). When considering Ga_2_O_3_ films, the band gap (*E*_g_) can be computed by utilizing the equation: α*hv* = *A*(*hv* – *E*_g_)^1/2^. Here, α signifies the absorption coefficient, *h* stands for Planck’s constant, *v* represents the incident light frequency, and *A* denotes the material-specific constant.^44−46^ For both the undoped and Sn doped Ga_2_O_3_ films, a band gap of 4.8 eV was observed.

**Figure 4:**
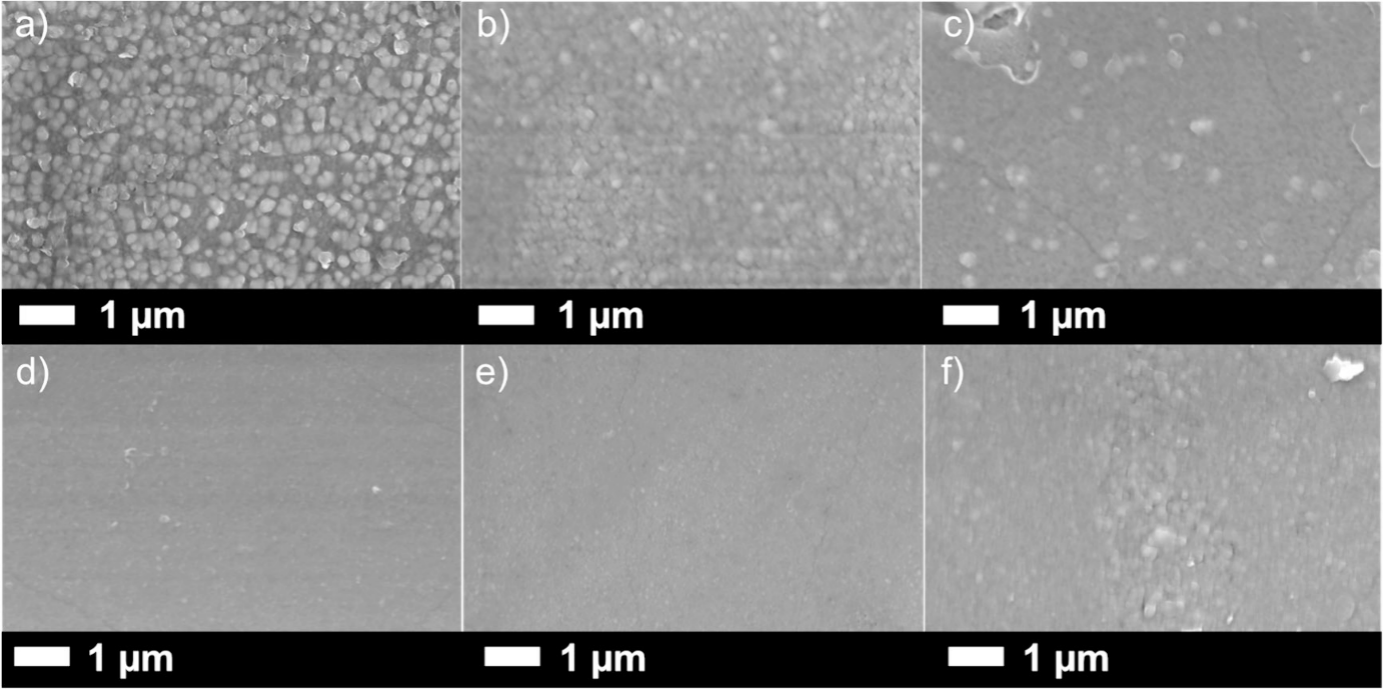
SEM images showing a faceted morphology for (a) 1.7 at%; (b) 2.5 at%; (c) 3.6 at%; (d) 3.9 at%; (e) 4.3 at%; (f) 6.2 at% Sn-doped Ga_2_O_3_ films annealed in air at 1000 °C for 12 h.

Figure 4 illustrates the surface morphology of Ga_2_O_3_ films at varying Sn doping concentrations. An obvious trend emerges as the Sn doping concentration increases: the grain size becomes more uniform, resulting in a smoother and flatter film surface. Specifically, when the Sn doping concentration remains below 2.3 at. %, a coexistence of relatively larger grains, ranging from 150 to 250 nm in diameter, and finer particles was observed on the film surface. However, upon reaching a Sn doping concentration of 2.5 at%, the surface morphology of the Ga_2_O_3_ film showed a higher degree of uniformity, with grain sizes primarily concentrated within the 100–150 nm range. At higher doping concentrations, the SEM images reveal the presence of fine cracks. These cracks are most likely due to the annealing step applied to the films to obtain the monoclinic phase of Ga_2_O_3_ or the alteration of internal stress within the film due to changes in grain size. The alteration in surface morphology observed in the films could potentially be attributed to the annealing process, a phenomenon seen in previous research.^47^ Upon deposition of Ga_2_O_3_ films onto sapphire substrates followed by annealing, grain-like structures emerged on the film surface, while internal grain boundaries were not discernible. These grain-like structures were identified to be associated with β-Ga_2_O_3_.

Concurrently, the cross-sectional analysis of the samples using SEM provides insights into the thickness of the Ga_2_O_3_ film deposited on the substrate (see ESI, Figure S1). As depicted in side-SEM results, the data suggest that the Sn doping concentration exerts minimal influence on the thickness of the Ga_2_O_3_ films, all of which fall within the range of 240–350 nm. Based on the side-view SEM results, it is evident that the doping of Sn induces a gradual granulation of the Ga_2_O_3_ microstructure, although obvious grain boundaries are not readily observed as seen in previous research.^47,48^

**Figure 5:**
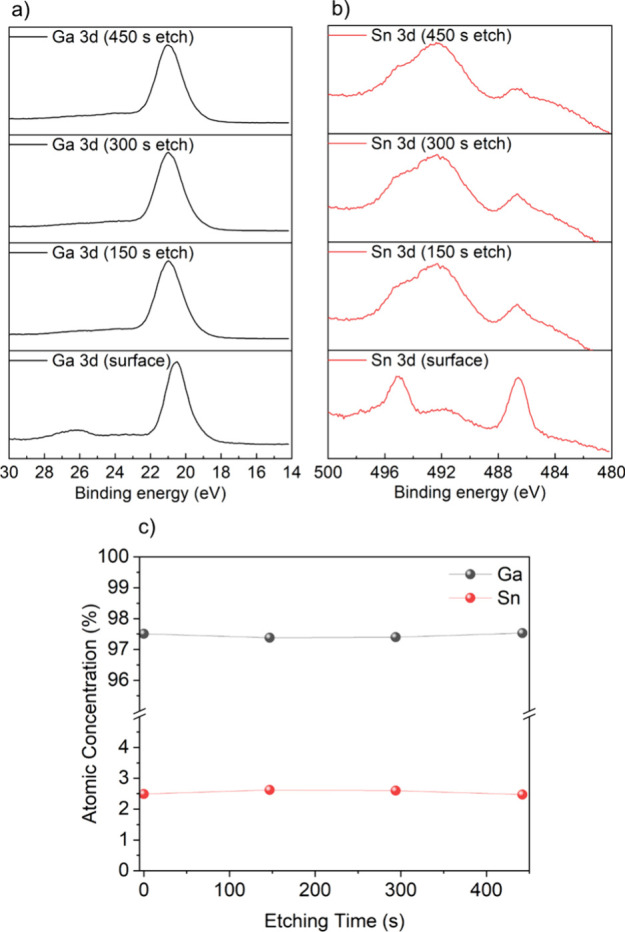
XPS of an 2.5 at% Sn-doped Ga_2_O_3_ film showing the (a) Ga 3d and (b) Sn 3d regions from the surface, 150, 300, and 450 s Ar etch to indicate the Sn dopant is present both on the surface and the bulk. (c) Trends in atomic concentration according to etching time.

To investigate the composition of the Ga_2_O_3_ film, X-ray photoelectron spectroscopy (XPS) analysis was conducted, and the results are presented in Figure 5. Fitting of the surface Ga 3d and Sn 3d scans reveal Ga to be in the 3+ (Ga 3d_5/3_ centered at 20.5 eV) and Sn in 4+ (Sn 3d_5/2_ centered at 486.4 eV) oxidation states for all the films. For the C 1s core level, the primary peak is centered at 284.8 eV, corresponding to the C–C bond. Additionally, two secondary peaks are seen at 286.8 and 289.1 eV, signifying the presence of the C–O and C=C bonds, respectively, which is consistent with the findings in the reference literature.^49^ The O 1s spectra were fit with three peaks. The principal component, situated at 530.9 eV, corresponds to the Ga–O bond, constituting the predominant component of the Ga_2_O_3_ thin film. Two minor peaks, at 530.2 and 532.5 eV, can be matched with literature values corresponding to Sn–O and C–O bonds, respectively.^50,51^ There is also the possibility of the existence of other O^2–^ adsorbed species, such as O–H bonds.

Depth-resolved XPS analysis was also conducted on the doped films. As illustrated in Figure 5, a discernible disparity in the chemical environment was evident between the surface of the sample and the interior of the film. On the sample surface, a small peak appeared on the left side of the Ga 3d signal peak, which corresponds to the O 2s signal peak. However, this peak disappeared after etching, indicating the possible presence of adsorbates, or surface hydroxides on the thin film. These substances were absent from the interior of the sample. Similar results were also mentioned in the study by Ming–Ming et al.^26^ The etching experiments also show a slightly higher Sn content in the interior of the thin film compared to that on its surface, possibly due to bulk segregation of the dopant. It should be noted that the differences in the Sn 3d peak shape seen at the surface and etched levels are due to preferential sputtering effects that readily take place during etching of metal oxides under high vacuum.

**Figure 6:**
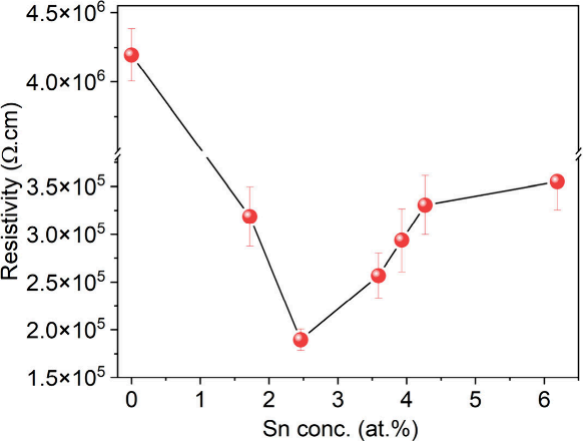
Electrical resistivity versus Sn atomic concentration for Sn-doped Ga_2_O_3_ films deposited on quartz via AACVD.

Figure 6 presents the relationship between the Sn doping content and the film resistivity of Ga_2_O_3_ films. The resistivity of the pristine Ga_2_O_3_ film measures approximately 4.2 × 10^6^ Ω cm. Notably, a pronounced reduction in resistivity was observed following Sn doping, with the film exhibiting its lowest resistivity of 1.90 × 10^5^ Ω·cm when the Sn concentration reached 2.5 at. %. The lowest resistivity was consistent with previous studies.^6^ Overall, Ga_2_O_3_ crystals exhibit superior electrical performance compared to Ga_2_O_3_ thin films, which typically manifest resistivity values exceeding 10^13^ Ω cm. Maria Isabel Pintor-Monroy et al.^6^ fabricated nanocrystalline thin films utilizing pulsed laser deposition (PLD) and molecular beam epitaxy (MBE) techniques, achieving a reduced resistivity of 2 × 10^5^ Ω cm. Zhiwei Li et al.^52^ employed the optical floating zone (OFZ) method to fabricate Ga_2_O_3_ thin films doped with Al elements, resulting in a resistivity of 1.5 × 10^12^ Ω cm. Wei Mi et al.^53^ utilized the metal organic chemical vapor deposition (MOCVD) technique to synthesize Sn-doped Ga_2_O_3_ thin films, thereby decreasing the resistivity to 5.5 × 10^12^ Ω cm, reaching 5.4 × 10^7^Ω cm with a 3 mol % Sn doping concentration.

Previous computational and analytical investigations have established that Sn doping enhances the concentration of free electrons without excessively diminishing their mobility within the material.^11,54−56^ Concurrently, Sn doping may induce alterations in the crystal structure of the material such as lattice distortion or the introduction of crystal defects. Inspection of the XRD results reveal that Sn-doped Ga_2_O_3_ thin films exhibit an expansion in the unit cell volume, ranging from 0.9% to 1.5%, when compared to pure Ga_2_O_3_.

## Conclusion

In summary, this study outlines the deposition process of Sn-doped Ga_2_O_3_ thin films on quartz substrates utilizing AACVD. With increasing dopant concentration a decrease in film resistivity was observed presumably due to increasing carrier density afforded by the Sn4+ substituting on Ga3+ sites in the Ga_2_O_3_ lattice. The lowest resistivity of 1.8 × 10^5^ Ω cm was achieved for the 2.5 at. % Sn:Ga_2_O_3_ sample, this was more than an order of magnitude lower than that seen for the nominally undoped Ga_2_O_3_ film (4.2 × 10^6^ Ω cm). Dopant concentrations beyond 2.5 at. % showed diminished electrical performance due to SnO_2_ phase separation taking place. The ultrawide band gap of Ga_2_O_3_ was maintained at 4.8 eV even after doping.

## Supporting Information Available

Side on SEM, fwhm data, XPS spectra of C 1s and O 1s, Hall effect measurements (PDF)
