# Supplementary material for: An Aerosol-Assisted Chemical Vapor Deposition Route to Tin-Doped Gallium Oxide Thin Films with Optoelectronic Properties
Source: ACS Appl Electron Mater. 2024 Aug 12;6(8):6085–91. doi: 10.1021/acsaelm.4c00973 (PMC11360363; doi:10.1021/acsaelm.4c00973)
Supplement: Supplementary file 1 — el4c00973_si_001.pdf [file el4c00973_si_001.pdf]

Supporting Information

An aerosol assisted chemical vapour deposition route to tin doped gallium oxide thin films with optoelectronic properties

Ruizhe Chen[a], Sanjayan Sathasivam[a,b] Joanna Borowiec[a] and Claire J Carmalt[a]*

*Corresponding authors

[a] Materials Chemistry Centre, Department of Chemistry, University College London, 20 Gordon Street, London WC1H 0AJ, UK

[b] School of Engineering, London South Bank University, London SE1 0AA, UK

E-mail: c.j.carmalt@ucl.ac.uk

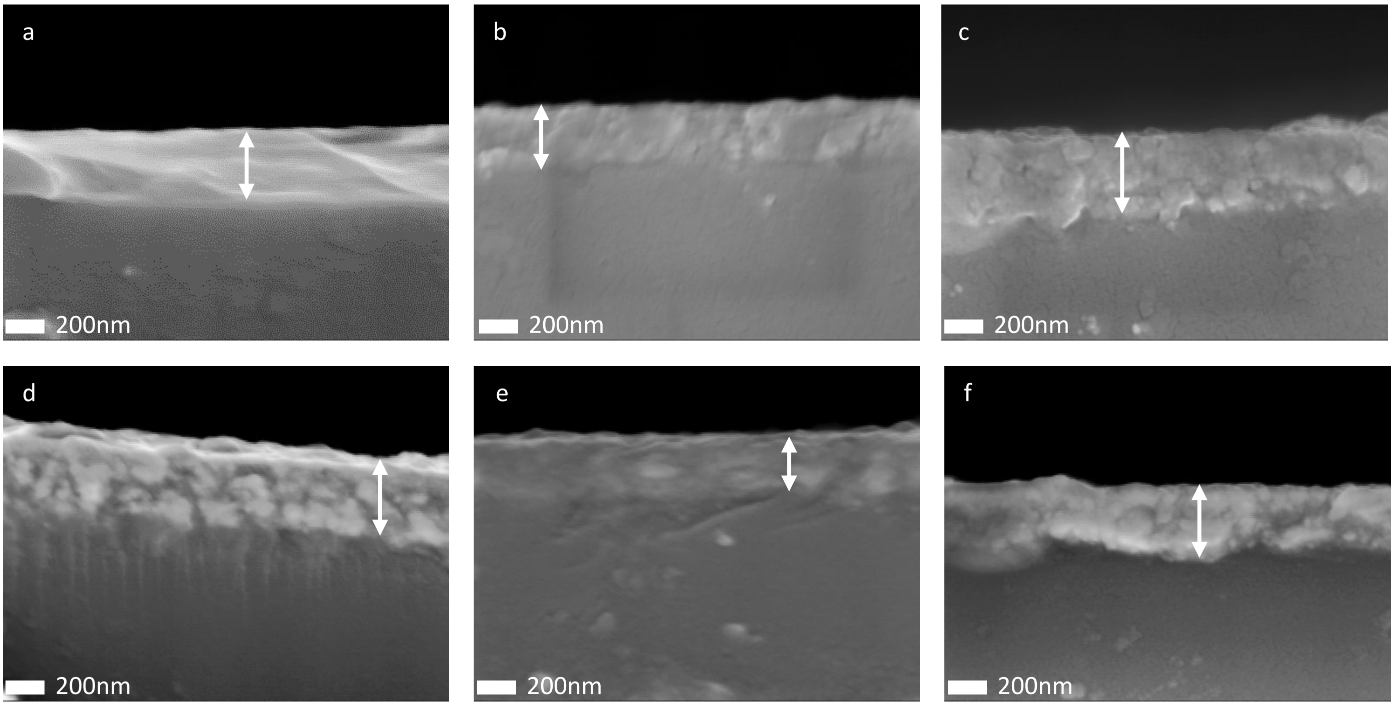

Figure S1. SEM side test morphology of different Sn concentration of Sn-doped Ga_2O_3: a) 1.7at%; b) 2.5at%; c) 3.6at%; d) 3.9at%; e) 4.3at%; f) 6.2at%.

Table S1: The FWHM of the representative -(202) XRD peak as an indication of the changing crystallinity of the Sn doped Ga2O3 AACVD films after annealing in air for 12 hours at 1000 °C.

Sn concentration (at.%)	FWHM (°)
0	0.202
1.7	0.174
2.5	0.162
3.6	0.192
3.9	0.311
4.3	0.277
6.2	0.221

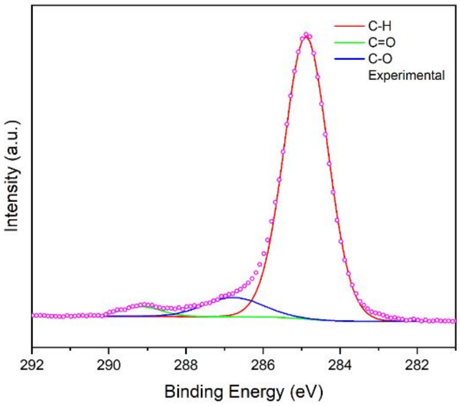

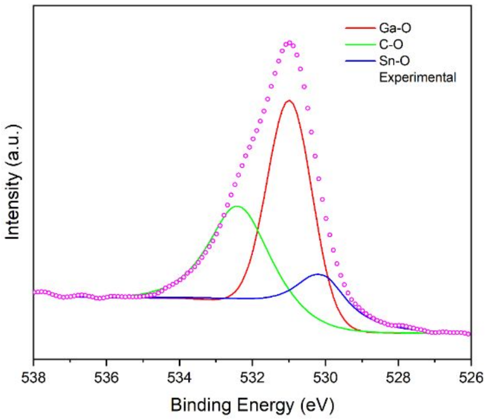

Figure S2: The fitted XPS spectra of representative C 1s signal (top) and the O 1s signal (bottom) for the Sn doped Ga_2O_3 films grown via AACVD and annealed in air at 1000 °C for 12 hours.

Table S2: The resistivity determined by Hall Effect instrument.

Sn Concentration	Film Thickness(nm)	Resistivity(Ωcm)
0 at.%	365.0	4.195×10^{6}
1.7 at.%	291.6	3.186×10^{5}
2.5 at.%	324.3	1.896×10^{5}
3.6 at.%	371.1	2.568×10^{5}
3.9 at.%	352.3	2.937×10^{5}
4.3 at.%	287.8	3.306×10^{5}
6.2 at.%	375.8	3.549×10^{5}
